# Drought-Induced Mortality in *Phoebe bournei* Seedlings: Interactive Effects of Hydraulic Failure and Carbon Starvation

**DOI:** 10.3390/plants15152294

**Published:** 2026-07-27

**Authors:** Meiling Gao, Xiaoshan Chen, Yang Mo, Jincheng Yang, Qian He, Yan Su, Quan Qiu

**Affiliations:** 1 Guangdong Key Laboratory for Innovative Development and Utilization of Forest Plant Germplasm, College of Forestry and Landscape Architecture, South China Agricultural University, Guangzhou 510642, China; 20243154020@stu.scau.edu.cn (M.G.); chenxs@stu.scau.edu.cn (X.C.); my1569@163.com (Y.M.); yangjincheng@stu.scau.edu.cn (J.Y.); heqian@scau.edu.cn (Q.H.); 2 Key Laboratory of National Forestry and Grassland Administration for Forest Therapy and Wellness, College of Forestry and Landscape Architecture, South China Agricultural University, Guangzhou 510642, China

**Keywords:** carbon starvation, drought, hydraulic failure, mortality mechanism, non-structural carbohydrates, *Phoebe bournei*, xylem conductivity

## Abstract

Drought stress is a major environmental factor limiting plant growth and distribution, with severe drought leading to plant mortality. This study investigates the physiological mechanisms underlying drought-induced mortality in one-year-old seedlings of the valuable timber tree species *Phoebe bournei* (Hemsl.) Yang, aiming to clarify the relative roles of hydraulic failure and carbon starvation. A 51-day controlled pot experiment was conducted to simulate progressive drought using 10 experimental groups (n = 6): a well-watered control and four drought treatment groups harvested at key physiological stages. Stage I (baseline) corresponded to a relative soil water content of approximately 89%. Stage II (photosynthetic cessation) was reached after approximately 15 days of water withholding, at a relative soil water content of approximately 60% and a predawn leaf water potential of approximately −3.4 MPa. Stage III (complete leaf wilting) was reached after approximately 36 days of water withholding. Stage IV (stem browning) occurred at a relative soil water content of approximately 18%, after approximately 45–51 days of water withholding. We systematically measured key physiological parameters, including leaf water potential, gas exchange parameters, the percentage loss of xylem conductivity in stems, and the concentrations of non-structural carbohydrates (including soluble sugars and starch) in different tissues. Results showed that stomatal conductance and net photosynthetic rate approached zero when leaf water potential fell to approximately −3.4 MPa. Stem percentage loss of xylem conductivity increased significantly with advancing drought, exceeding 75% at complete leaf wilting and reaching over 98% at stem browning, reflecting a near-complete loss of xylem hydraulic conductance. Concurrently, non-structural carbohydrate concentrations underwent transient accumulation during early drought, reflecting sink-limited carbon dynamics, followed by progressive depletion. Notably, partial non-structural carbohydrate reserves persisted even at the stem browning stage, suggesting that these reserves may have become physically inaccessible or metabolically unavailable rather than entirely exhausted. The findings point to a tightly coupled, sequential interaction between hydraulic failure and carbon starvation across the drought progression. The findings will provide a scientific basis for evaluating drought tolerance, informing adaptive management practices, and ensuring the sustainable cultivation of *P. bournei* under future climate scenarios.

## 1. Introduction

Climate change is intensifying the frequency, severity, and duration of drought events, threatening forest ecosystem structure and function and driving widespread tree decline and mortality [1,2,3]. Elucidating the physiological mechanisms of drought-induced tree mortality has therefore become a central focus of global change ecology [4,5,6]. Two principal hypotheses have been advanced to explain this phenomenon [7,8,9]. The hydraulic failure hypothesis holds that mortality arises from catastrophic collapse of the xylem water transport system, wherein intensifying soil drought drives cavitation and embolism propagation that progressively reduce hydraulic conductivity until canopy water supply fails [10,11,12]. The carbon starvation hypothesis attributes mortality to disrupted carbon metabolism, whereby drought-induced stomatal closure suppresses photosynthesis and forces dependence on stored non-structural carbohydrates (NSC), whose progressive depletion, when consumption outpaces replenishment, leads to metabolic crisis and death [13,14,15]. These mechanisms likely interact, as hydraulic failure can impair phloem transport and accelerate carbon depletion, while carbon starvation may compromise the metabolic repair of hydraulic damage [16,17]. The relative importance and temporal sequence of the two mechanisms remain species-specific and actively debated, particularly for angiosperms, whose distinct xylem anatomy confers fundamentally different hydraulic vulnerabilities relative to conifers.

*Phoebe bournei* (Lauraceae) is an evergreen broad-leaved tree endemic to China, distributed across the subtropical regions of Fujian, Jiangxi, Hunan, Guangdong, Guangxi, and Guizhou provinces [18,19]. It is a valuable timber species prized for its durable, finely grained, golden-yellow wood, and is listed as a national Class II protected plant in China. The species is sensitive to deviations in hydrothermal conditions, making it an ideal model for studying drought vulnerability in subtropical evergreen trees [20]. Anatomically, Lauraceae species including *P. bournei* characteristically possess large-diameter xylem vessels with simple perforation plates, conferring high hydraulic conductivity but also intrinsically high vulnerability to drought-induced embolism [21,22]. This reflects the classic hydraulic safety-efficiency trade-off, wherein wide vessels maximize water transport under favorable conditions but are inherently susceptible to air-seeding and embolism under water deficit [11,23]. Compared to conifers with torus-margo pit membranes that effectively compartmentalize embolisms, or diffuse-porous angiosperms with smaller vessel diameters, *P. bournei*’s vessel architecture may predispose it to rapid hydraulic failure under severe drought, providing a mechanistic basis for the hypothesis that hydraulic failure initiates the mortality cascade in this species. Previous physiological studies of *P. bournei* have mainly addressed moderate water deficit. Ge et al. reported that seedlings mount active osmotic adjustment and antioxidant defense under mild to moderate drought but exhibit steep physiological decline under severe stress [18]. Wu et al. demonstrated that *P. bournei* adopts a relatively anisohydric strategy, maintaining high transpiration rates under declining leaf water potential, a behavior that may compound hydraulic risk [21]. Frequent seasonal droughts in South China increasingly threaten both plantation establishment and natural regeneration of this species [24,25]. Despite these advances, critical knowledge gaps remain when comparing *P. bournei* with other well-studied tree species. First, the relative roles and interaction of hydraulic failure and carbon starvation during drought-induced mortality have been studied predominantly in conifers and temperate angiosperms [9,13], whereas comparable data for subtropical evergreen broad-leaved trees with large-diameter vessels, such as *P. bournei*, are absent. Given that *P. bournei* possesses wider xylem conduits and higher intrinsic hydraulic vulnerability than most previously studied species [21,22], whether the temporal sequence and coupling strength of the two mechanisms differ from established patterns remains an open question. Second, lethal physiological thresholds, including the critical levels of percentage loss of xylem conductivity (PLC) and leaf water potential beyond which recovery is impossible, have been quantified for several conifers and temperate angiosperms [26,27], yet corresponding values for *P. bournei* have not been determined. This limits the ability to assess whether this subtropical species is disproportionately vulnerable relative to better-characterized taxa. Third, in some drought-tolerant conifers, substantial NSC reserves persist at mortality and are thought to reflect active metabolic downregulation rather than resource exhaustion [7,14]; whether *P. bournei*, with its distinct xylem anatomy and carbon allocation strategy, exhibits a similar pattern of carbon reserve retention or instead undergoes more rapid and complete NSC depletion is unknown.

Based on the above considerations, we formulated three testable hypotheses: (H_1_) hydraulic failure and carbon starvation in *P. bournei* seedlings do not operate as independent, hierarchically ordered mechanisms, but rather as interacting components of a coupled physiological cascade in which each process influences the other; (H_2_) the interaction between these two mechanisms can be characterized as a three-phase progression of coordination, amplification, and terminal decline, with the relative contribution of each mechanism dynamically shifting as drought progresses; and (H_3_) there exists a quantifiable physiological threshold beyond which drought-induced damage becomes irreversible, such that re-watering can no longer rescue the seedlings. To test these hypotheses, we conducted a controlled pot experiment with the following specific objectives: (1) to characterize the temporal sequence and coupling dynamics of hydraulic dysfunction and carbon depletion under progressive drought stress across four well-defined physiological stages, and (2) to identify key physiological thresholds (e.g., leaf water potential, percentage loss of xylem conductivity) associated with mortality.

## 2. Materials and Methods

### 2.1. Experimental Site Description

The experiment was conducted at the South China Agricultural University nursery (23°9′20″ N, 113°21′13″ E). This region is characterized by a typical subtropical monsoon climate, with a mean annual temperature of 23.1 °C and an annual precipitation of 2536.7 mm (Guangzhou Meteorological Bureau, 2024). The nursery site provides adequate ventilation and sufficient sunlight. Furthermore, it is equipped with rain-shelter plastic greenhouses, creating an environment suitable for growing tree saplings.

### 2.2. Experimental Materials

We utilized healthy, uniform one-year-old seedlings of *P. bournei* as experimental material, with initial average seedling height and ground diameter of 63.58 ± 6.06 cm and 2.14 ± 0.31 mm (mean ± SD), respectively. The seedlings were cultivated in pots with an upper diameter of 22.5 cm, a lower diameter of 18 cm, and a height of 21 cm, filled with a growth substrate composed of a 3:1 volume ratio of yellow subsoil and peat soil, yielding a total mass of approximately 5 kg per pot. The substrate’s physicochemical properties were thoroughly characterized: chemically, it exhibited a total nitrogen content of 0.72 g kg^−1^, total phosphorus content of 0.22 g kg^−1^, organic content of 21.35 g kg^−1^, alkali-hydrolyzable nitrogen content of 98.64 mg kg^−1^, available phosphorus content of 4.14 mg kg^−1^, and a pH value of 5.36; physically, it demonstrated a bulk density of 1.16 g cm^−3^ and a field capacity of 30.27%. Following a four-month acclimatization period under consistent, well-watered conditions, the seedlings established robust growth, ensuring their suitability for subsequent experimental treatments.

### 2.3. Experimental Design and Methods

The experimental design comprised two treatments: a control group (CK) maintained under well-watered conditions throughout the study, and a drought treatment group subjected to progressive soil drying to simulate natural drought-induced mortality processes. The drought treatment was divided into four critical stages based on physiological milestones: Stage I (baseline, one day before trial initiation), Stage II (when leaf net photosynthetic rate declined to zero), Stage III (at 100% leaf wilting), and Stage IV (upon complete stem browning) (Table 1). For each stage except Stage I, a parallel re-watering group and a control group were established to assess potential recovery post-drought, serving as a verification of plant viability. Measurements of leaf gas exchange parameters and leaf water potential were conducted non-destructively on the same seedlings at three-day intervals throughout the experimental period. In contrast, hydraulic conductivity measurements and tissue collection for non-structural carbohydrate analysis involved destructive sampling at each stage endpoint. Each stage constituted an independent group, with a total of ten experimental groups: baseline stage (n = 6), zero photosynthesis stage (drought group, control group and re-watering group, each n = 6), leaf wilting stage (drought group, control group and re-watering group, each n = 6), and stem browning stage (drought group, control group and re-watering group, each n = 6). At each endpoint, plants from both the CK and drought groups were photographed, followed by measurements of leaf gas exchange parameters and leaf water potential. Subsequently, destructive sampling was conducted to determine fresh weights of roots, stems, and leaves, and to assess hydraulic conductivity. The collected plant tissues were then oven-dried at 105 °C for 1 h to deactivate enzymes, followed by drying at 65 °C to constant weight for dry mass quantification and non-structural carbohydrate analysis. Concurrently, re-watering groups were irrigated at their respective stage endpoints and monitored every five days for one month to record any signs of recovery, such as leaf re-greening.

#### 2.3.1. Soil Water Content Measurement

Soil volumetric water content (VWC) was measured at approximately 10 cm depth within the root zone using a portable time-domain reflectometry (TDR) soil moisture meter (TDR 300, Spectrum Technologies, Inc., Aurora, IL, USA) every three days [28]. Soil bulk density was determined concurrently by the cutting ring method. To ensure measurement accuracy, gravimetric soil water content was determined on parallel soil samples collected in aluminum boxes at each measurement time for calibration of the TDR readings.

#### 2.3.2. Gas Exchange Parameters

Leaf gas exchange parameters were measured with a portable photosynthesis system (LI-6400; LI-COR Inc., Lincoln, NE, USA) from the onset of drought until the net photosynthetic rate reached zero. Measurements were taken daily from 09:30 to 11:00 on fully developed south-facing leaves at the fourth to fifth branch whorls. For each plant, three randomly selected leaves were assessed as replicates. The instrument maintained a photon flux density of 1200 μmol m^−2^ s^−1^, a CO_2_ concentration of 400 μmol mol^−1^, and a flow rate of 500 μmol s^−1^. Recorded parameters included net photosynthetic rate (Pn), stomatal conductance (Cond). Before logging data, leaves equilibrated in the cuvette until photosynthetic parameters stabilized. Measurements were taken at three-day intervals from the initiation of the water-withholding treatment, coinciding with the days scheduled for leaf water potential determinations.

#### 2.3.3. Water Potential

Leaf water potential (Ψ_leaf_) was measured using a digital portable plant water potential pressure chamber (Model 1505D; PMS Instrument Company, Albany, OR, USA). For each plant, three intact, fully expanded, healthy, and uniformly sized leaves were selected from the mid-crown region, and their measurements were averaged to represent the plant’s water status. Predawn measurements (Ψ_pd_) were taken between 04:00 and 06:00, before sunrise, to approximate soil water potential at equilibrium. Midday measurements (Ψ_md_) were taken between 12:00 and 14:00, representing the maximum daily water stress. Measurements were conducted every three days from the initiation of water withholding, synchronized with gas exchange measurements.

#### 2.3.4. Hydraulic Conductivity (*K*_h_)

During destructive sampling of seedlings, 10 cm stem segments were collected by making perpendicular cuts underwater to prevent air entry into the xylem vessels. The stem segment length of 10 cm was selected because it exceeds the reported average vessel length of Lauraceae species (typically 2–8 cm), thereby minimizing open-vessel artifacts that could otherwise lead to overestimation of hydraulic conductivity. To further reduce potential artifacts, the following precautions were implemented: (1) stem segments were collected from the basal portion of the main stem where vessel lengths are typically shorter; (2) the underwater cutting technique prevented air entry into xylem vessels during sample preparation; and (3) vacuum perfusion with degassed water prior to maximum conductivity measurement removed any residual emboli, providing a reliable baseline for PLC calculation. The specific experimental methods are as follows: The bark at both ends was carefully removed to expose the underlying xylem tissue, which was immediately wrapped with parafilm to create a sealed connection. Following underwater re-cutting to ensure embolism-free samples, the segments were connected to silicone tubing with diameters matching the stem size, establishing an airtight interface with the water-conduction system [29]. The experimental apparatus was prepared by pouring degassed deionized water into a 500 mL graduated cylinder. Any residual air bubbles within the system were eliminated via syringe purging before measurements. The stem segments were positioned horizontally on the laboratory bench, with the water surface maintained 1 m above the segment plane. A pipette was connected to the silicone tubing to facilitate flow measurement; triplicate replicates were performed for each plant sample, and results were averaged for statistical reliability. The initial hydraulic conductivity (*K*_0_) was measured by utilizing the hydraulic pressure generated by a water column in a graduated cylinder to drive water flow through the excised stem segment. After allowing the system to stabilize for 5 min to achieve steady-state flow conditions, the time required for a specific volume of water to pass through the segment was recorded. This flow rate measurement was used to calculate *K*_0_ based on the pressure differential and segment dimensions. Following the initial measurement, the stem segment underwent 30 min of vacuum perfusion with degassed water to remove embolisms and restore maximum hydraulic capacity. This perfusion process was conducted under partial vacuum to facilitate the removal of air bubbles from the xylem vessels. The maximum hydraulic conductivity (*K*_max_) was then determined using the same flow measurement protocol. The formulas used for calculating hydraulic conductivity and the percentage loss of hydraulic conductivity are as follows:

(1) $$K_{h}=F\cdot L/∆P$$

(2) $$PLC=\left(1-\frac{K_{0}}{K_{max}}\right)$$

where *K*_h_ is the hydraulic conductivity, *F* is the water flow rate per unit time, *L* is the length of the stem segment, ∆*P* represents the pressure difference generated by the height difference between the meniscus of the graduated cylinder and the horizontal plane of the stem segment; PLC is the percentage loss rate of hydraulic conductivity, *K*_0_ is the initial hydraulic conductivity, and *K*_max_ is the maximum hydraulic conductivity.

#### 2.3.5. Non-Structural Carbohydrate Concentration

NSC concentrations (soluble sugars and starch) were quantified following the anthrone colorimetric method [30]. Pre-dried plant samples weighing 0.2 g were precisely weighed and transferred to 10 mL centrifuge tubes. The initial extraction involved adding 7 mL of 80% ethanol solution, followed by incubation in an 80 °C water bath for 30 min to facilitate sugar solubilization. After centrifugation, the supernatant was carefully collected, and the residual pellet underwent two additional extraction cycles using 6 mL of 80% ethanol solution each time, with centrifugation performed after each extraction. All collected supernatants were combined to a final volume of 50 mL in a volumetric flask, yielding the soluble sugar test solution. The remaining pellet was subsequently processed for starch quantification through gelatinization and acid hydrolysis. After adding 3 mL of distilled water, the samples were gelatinized in a boiling water bath for 15 min. Sequential extractions were performed using 2 mL of 9.2 mol L^−1^ perchloric acid, followed by 4.6 mol L^−1^ perchloric acid, and finally distilled water, with each extraction step followed by centrifugation. The combined extracts were diluted to 50 mL in a volumetric flask to prepare the starch test solution. For spectrophotometric determination, 1 mL of test solution was combined with 1 mL of distilled water, 0.5 mL of 1:50 anthrone-ethyl acetate solution, and 5 mL of concentrated sulfuric acid in a 10 mL stoppered graduated cylinder. The mixture was thoroughly vortexed and incubated in a boiling water bath for precisely 4 min. After cooling to room temperature, 0.2 mL of the reaction mixture was transferred to a microplate reader-compatible plate using a precision pipette. Absorbance measurements were conducted at 685 nm to quantify carbohydrate concentrations using established calibration curves.

### 2.4. Data Processing

Data were initially organized in Microsoft Excel 2019. All statistical analyses were performed using SPSS 27. Prior to parametric testing, the assumptions of normality and homogeneity of variances were verified using the Shapiro Wilk test and Levene’s test, respectively. For variables meeting both assumptions, one-way analysis of variance (ANOVA) was used to assess differences among drought stages and between treatments, followed by Tukey’s honestly significant difference (HSD) post hoc test for multiple comparisons. For pairwise comparisons between two groups (e.g., drought vs. control at a single stage), independent-samples *t*-tests were applied. Biological replicates consisted of six independent seedlings (n = 6) per treatment group. For gas exchange and leaf water potential measurements, three technical replicates (three leaves per seedling) were averaged to obtain a single representative value per individual plant prior to statistical analysis, ensuring that the experimental unit for all hypothesis tests was the individual seedling. All *p*-values from multiple comparisons were adjusted using the Tukey correction. When data did not meet the assumptions of parametric tests after transformation, non-parametric alternatives were employed. Statistical significance was set at *p* < 0.05. Graphs were generated using Origin 2025. All data in the text and figures are presented as mean ± standard deviation (SD).

## 3. Results

### 3.1. Response Characteristics of Phenotypic Parameters Under Drought Stress

**Figure 1:**
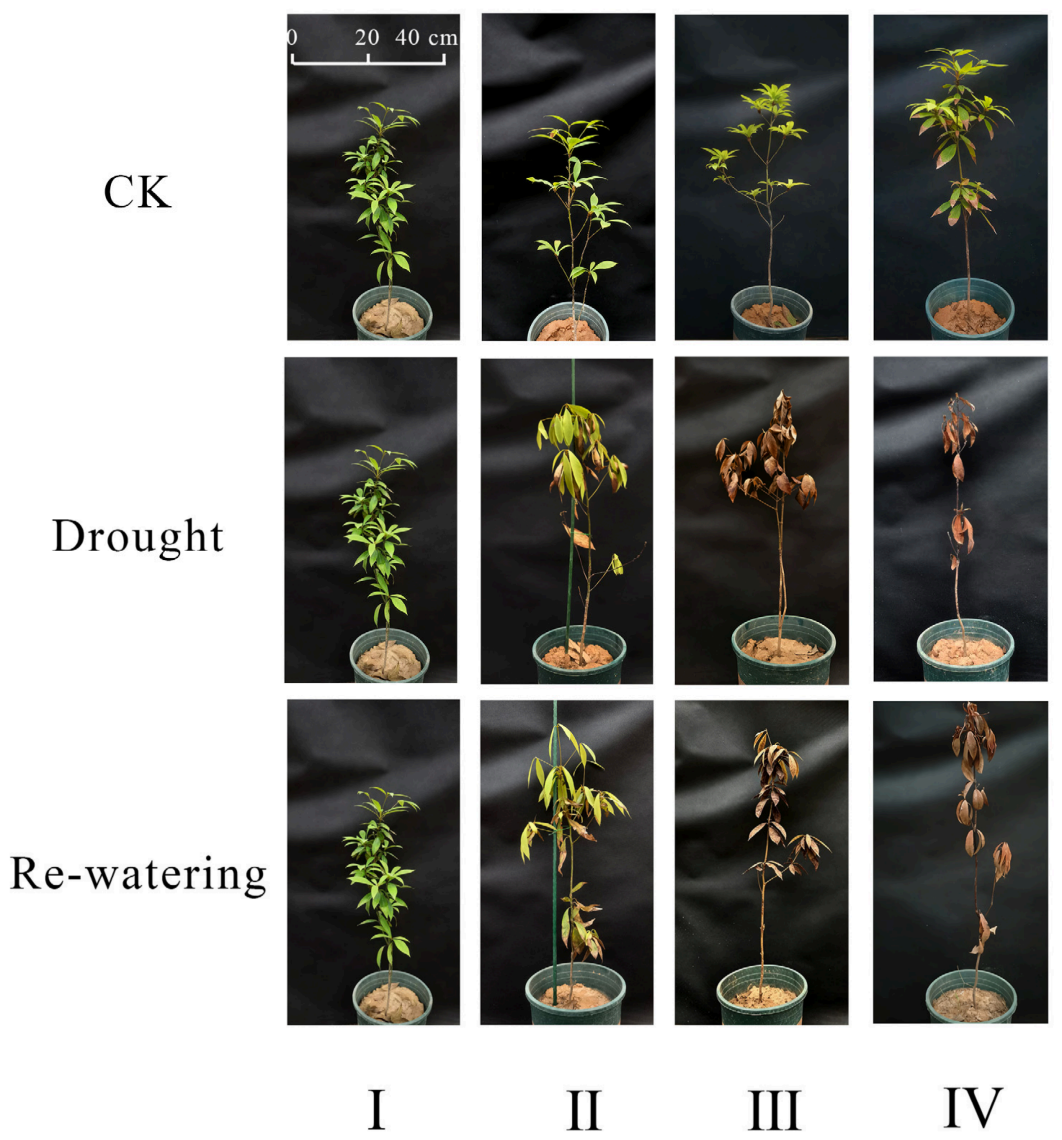
Growth status of *Phoebe bournei* seedlings under different conditions. (**I**) The day before the experiment began; (**II**) the net photosynthetic rate in the treated group was zero; (**III**) all leaves in the treated group wilted completely; (**IV**) the stem segments in the treated group turned brown and died.

Progressive drought treatment induced marked, stage-specific phenotypic changes in *P. bournei* seedlings relative to continuously well-watered controls (Figure 1). At Stage II (photosynthetic cessation), initial visible symptoms included leaf margin curling and slight chlorosis in approximately 30–50% of leaves per plant, with symptoms concentrated in older, lower-canopy leaves. By Stage III (complete leaf wilting), 100% of leaves exhibited severe wilting with pronounced desiccation and browning of leaf tips and margins, and leaf angles declined to near-vertical drooping positions, reflecting complete loss of turgor. At Stage IV (stem browning), stem tissues turned visibly brown with wrinkled bark texture, all leaves were completely desiccated and brittle to the touch, and the entire plant exhibited the morphological hallmarks of death. The re-watering experiment revealed clear, stage-dependent recovery thresholds. Seedlings re-watered at Stage II demonstrated physiological recovery: wilted leaves gradually regained turgidity within 5–10 days, defoliated plants regenerated new axillary buds within 15 days, and new leaf production was observed by day 20–30. The survival rate at this stage was 83% (5/6 seedlings). Seedlings re-watered at Stage III showed partial recovery with a survival rate of approximately 33% (2/6 seedlings). No seedlings re-watered at Stage IV showed any signs of recovery; survival rate was 0% (0/6).

### 3.2. Response Characteristics of Soil Water Content Under Drought Stress

**Figure 2:**
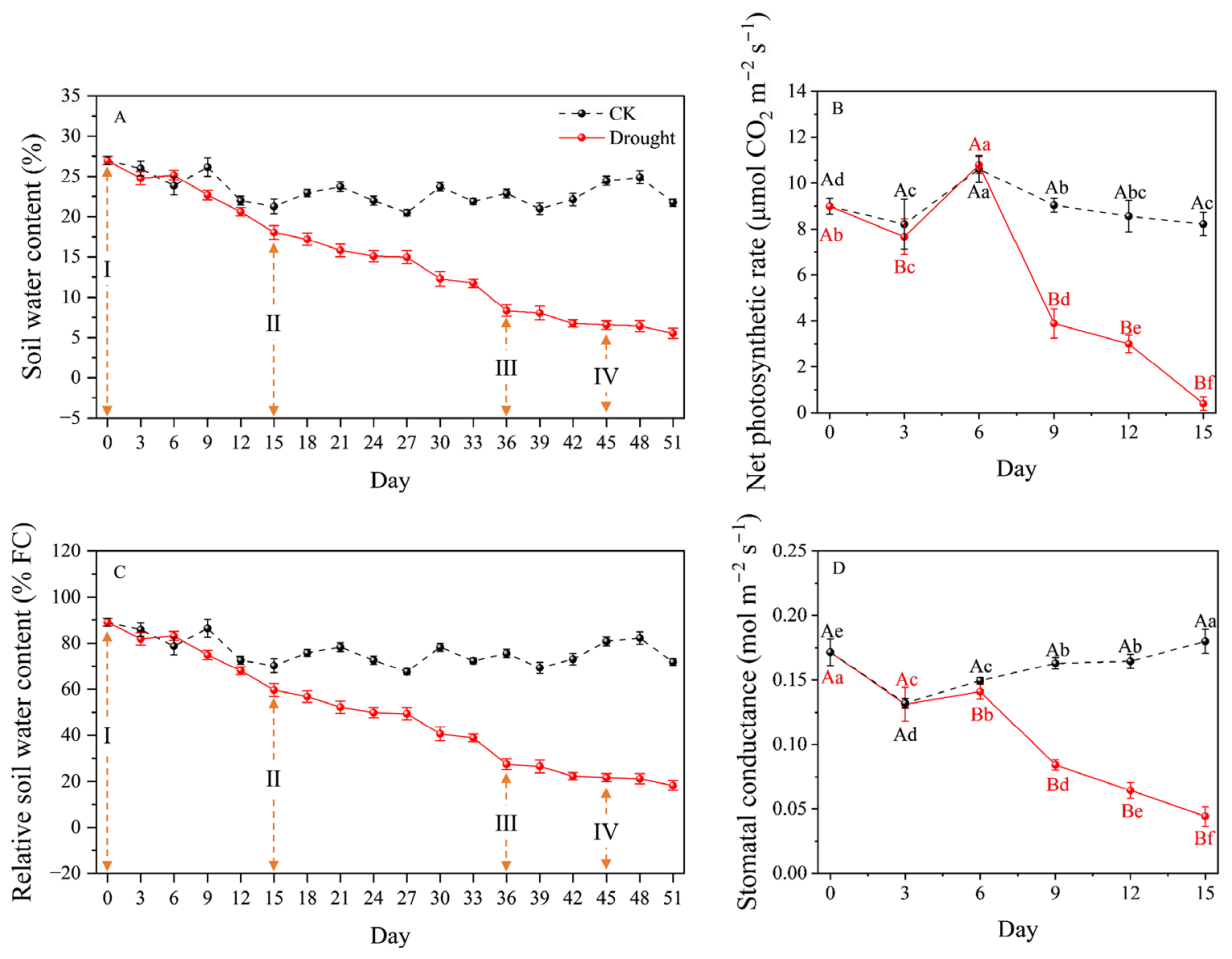
Changes in soil water content and relative soil water content during drought stress, and gas exchange parameters from the beginning of the experiment to zero net photosynthetic rate. (**A**) Soil water content; (**B**) net photosynthetic rate; (**C**) relative soil water content; (**D**) stomatal conductance. Different capital letters indicate significant differences between treatments at the same stage, while different lowercase letters indicate significant differences between stages within the same treatment (*p* < 0.05).

Experimental data revealed distinct patterns in relative soil water content (RSWC) between control and drought-treated groups throughout the 51-day experimental period (Figure 2). The control group maintained stable hydration conditions, with RSWC fluctuations ranging from 67.64% to 89.05% (mean ± SD: 89.05 ± 1.66% at day 0 to 71.86 ± 1.48% at day 51), confirming consistent water availability supporting normal physiological function. In contrast, drought-treated specimens exhibited a progressive dehydration pattern characterized by three distinct physiological phases.

During the early stress phase (days 0–9), RSWC declined from 89.05 ± 1.66% to 74.90 ± 1.95%, with an average daily reduction rate of 1.57%, suggesting active stomatal regulation and initial water-conservation mechanisms. The critical mid-stress phase (days 12–18) showed accelerated water loss from 68.07 ± 1.68% to 56.82 ± 2.49% (daily reduction: 1.88%), with day 15 recording 59.66 ± 2.82% RSWC—approximately 60% of field capacity. This threshold coincided precisely with the complete cessation of photosynthetic activity, indicating a fundamental physiological transition. The terminal stress phase (days 21–51) demonstrated catastrophic water depletion from 52.21 ± 2.63% to 18.23 ± 2.14% (daily reduction: 1.13%), with a stabilization period during days 45–51, consistent with failure of final osmotic adjustment. The ultimate RSWC value of 18.23% at plant mortality falls below the minimum threshold required for plasma membrane integrity maintenance (typically 20–30%), a level associated with ion leakage and cellular death. This progressive hydration failure demonstrates the critical relationship between soil water availability and physiological function in drought-induced mortality.

### 3.3. Response Characteristics of Hydraulic Under Drought Stress

#### 3.3.1. Leaf Water Potential

**Figure 3:**
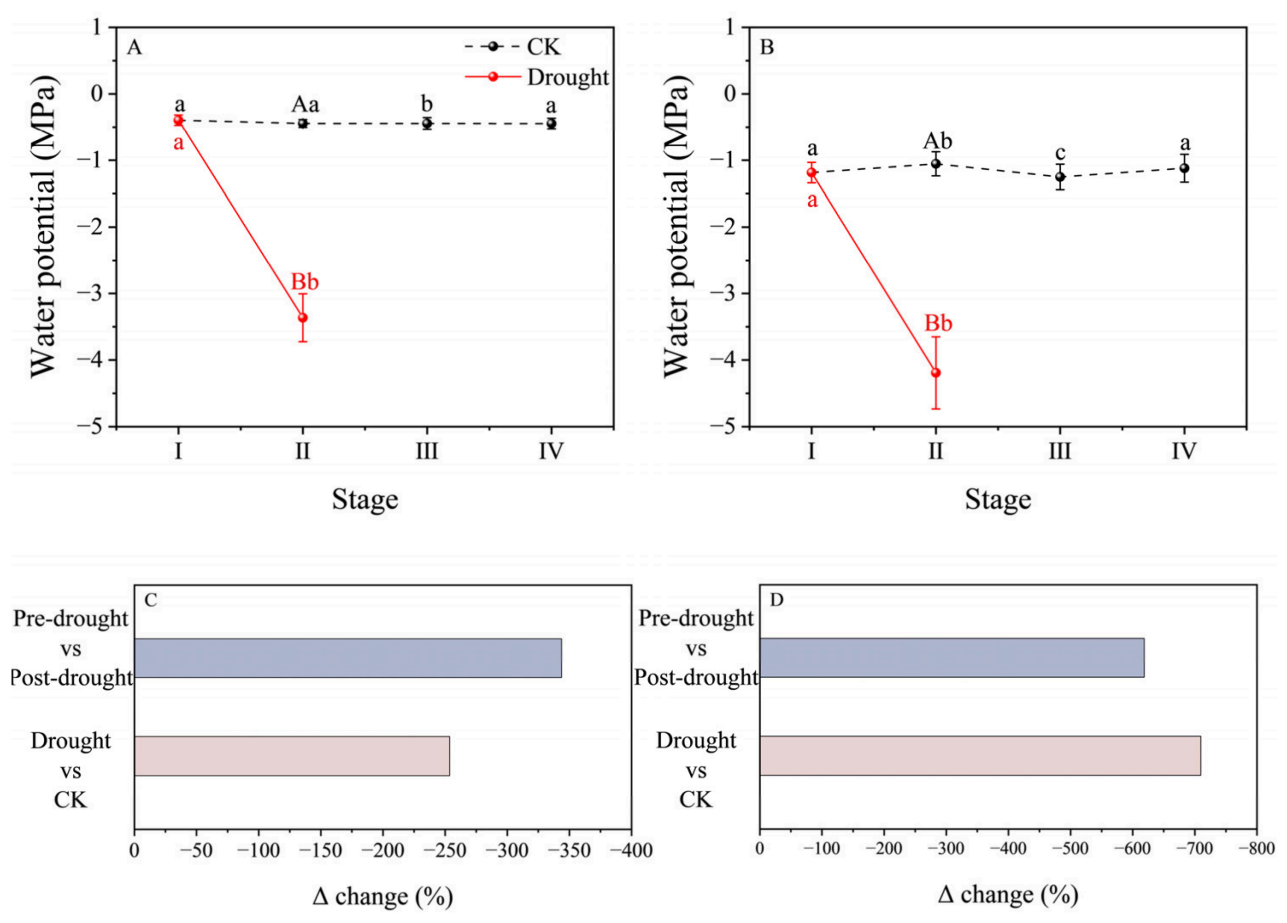
Leaf water potential changes under drought stress. (**A**) Predawn leaf water potential; (**B**) leaf water potential at noon; (**C**) changes in the predawn leaf water potential of the drought-stressed group relative to the initial value and the concurrent control group at different stages; (**D**) changes in the midday leaf water potential of the drought-stressed group relative to the initial value and the concurrent control group at different stages. Positive values indicate an increase, while negative values indicate a decrease. Stage III and stage IV values exceeded the instrument measurement range (<−5 MPa). Different capital letters indicate significant differences between treatments at the same stage, while different lowercase letters indicate significant differences between stages within the same treatment (*p* < 0.05).

Experimental observations revealed distinct diurnal patterns in leaf water potential between control and drought-treated *P. bournei* seedlings (Figure 3). Well-watered control plants maintained stable hydraulic status, with predawn measurements of −0.40 MPa and midday values of −1.19 MPa, showing significant diurnal variation (*p* < 0.05) attributable to nocturnal hydraulic recovery through reduced transpirational water loss and compensatory root water uptake. Drought-imposed conditions triggered progressive hydraulic degradation across distinct stress phases. During the zero-photosynthetic phase (II), water potential values declined to critical levels of −4.19 MPa at midday and −3.37 MPa at predawn, representing statistically significant reductions compared to contemporaneous controls (*p* < 0.05). These measurements indicate severe impairment of hydraulic conductivity, coinciding with complete cessation of photosynthesis. Advanced drought stages manifested catastrophic hydraulic failure beyond instrument detection capabilities. At full leaf wilting (III) and stem browning stages (IV), water potential values exceeded the measurement threshold of −5 MPa, preventing quantitative assessment. This instrumental limitation indicates severe cellular dehydration and points to a water potential depression beyond measurable limits. Such profound hydraulic collapse reflects comprehensive vascular embolism and the complete breakdown of water-transport systems, representing a fundamental physiological mechanism in drought-induced mortality.

#### 3.3.2. Hydraulic Conductivity

**Figure 4:**
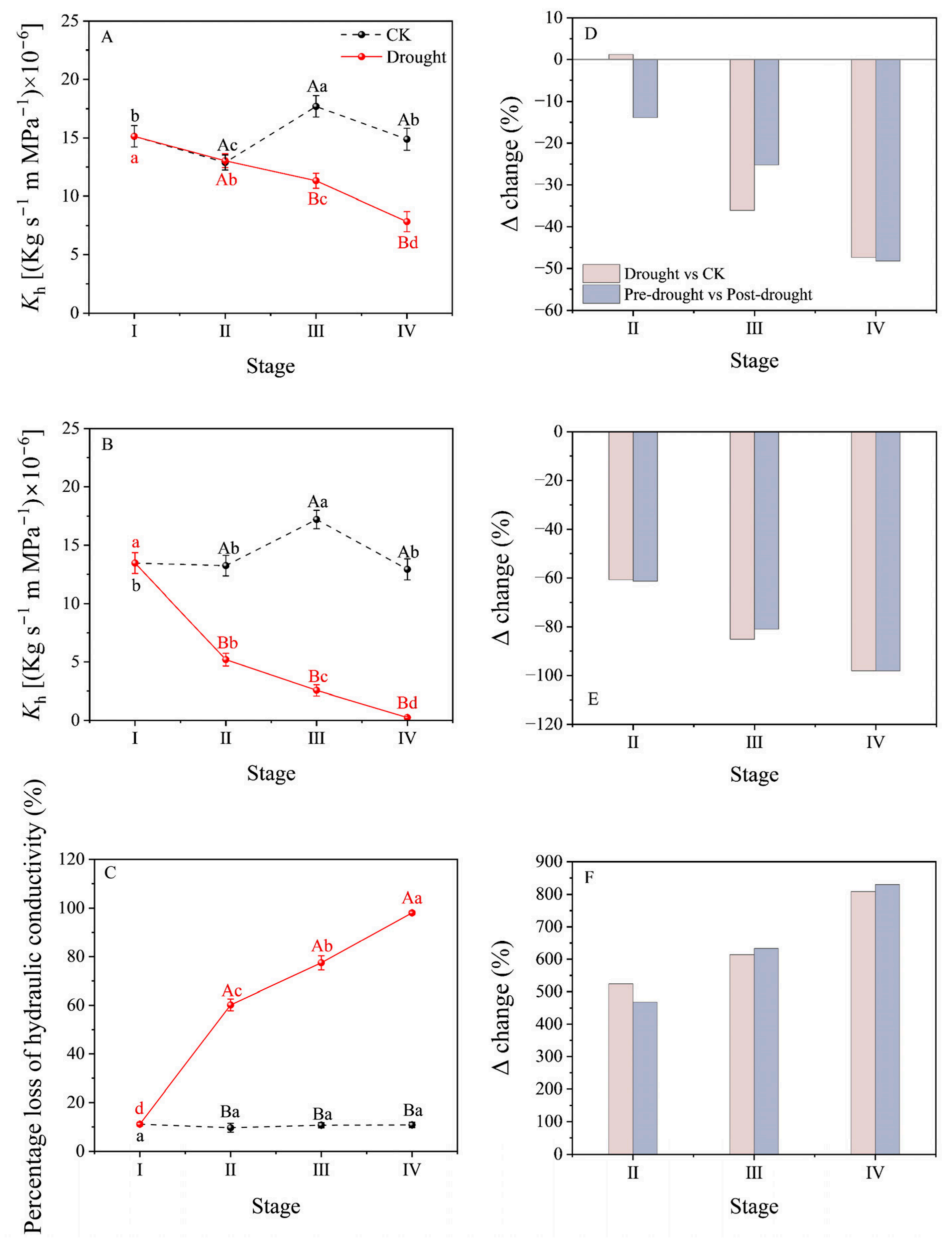
Changes in hydraulic conductivity and percent loss of hydraulic conductivity at various stages of *P. bournei*. (**A**) Maximum hydraulic conductivity; (**B**) initial hydraulic conductivity; (**C**) percentage loss of hydraulic conductivity; (**D**) changes in the maximum hydraulic conductivity of the drought-stressed group relative to the initial value and the concurrent control group at different stages; (**E**) changes in the initial hydraulic conductivity of the drought-stressed group relative to the initial value and the concurrent control group at different stages; (**F**) changes in the PLC of the drought-stressed group relative to the initial value and the concurrent control group at different stages. Positive values indicate an increase, while negative values indicate a decrease. Different capital letters indicate significant differences between treatments at the same stage, while different lowercase letters indicate significant differences between stages within the same treatment (*p* < 0.05).

Drought treatment significantly impaired stem hydraulic conductivity, resulting in reduced conductivity values and increased percentage loss of conductivity (PLC) (Figure 4). Control plants exhibited substantial fluctuations in initial hydraulic conductivity across phases II to IV, whereas drought-treated specimens showed a consistent decline in this parameter. The initial hydraulic conductivity of drought-treated plants was significantly lower than that of controls at all critical physiological stages. Maximum hydraulic conductivity followed a pattern similar to initial conductivity, with control plants showing variable values and drought-treated plants exhibiting a progressive decline. The PLC increased continuously throughout the experimental period, reaching 60% at the zero photosynthetic rate phase, 77% at complete leaf wilting, and culminating at 98% during the stem browning phase. This progressive decline in conductivity demonstrates the cumulative damaging effects of drought stress on the vascular system’s water-transport capacity.

### 3.4. Response Characteristics of Non-Structural Carbohydrates Under Drought Stress

#### 3.4.1. Soluble Sugar Concentration

**Figure 5:**
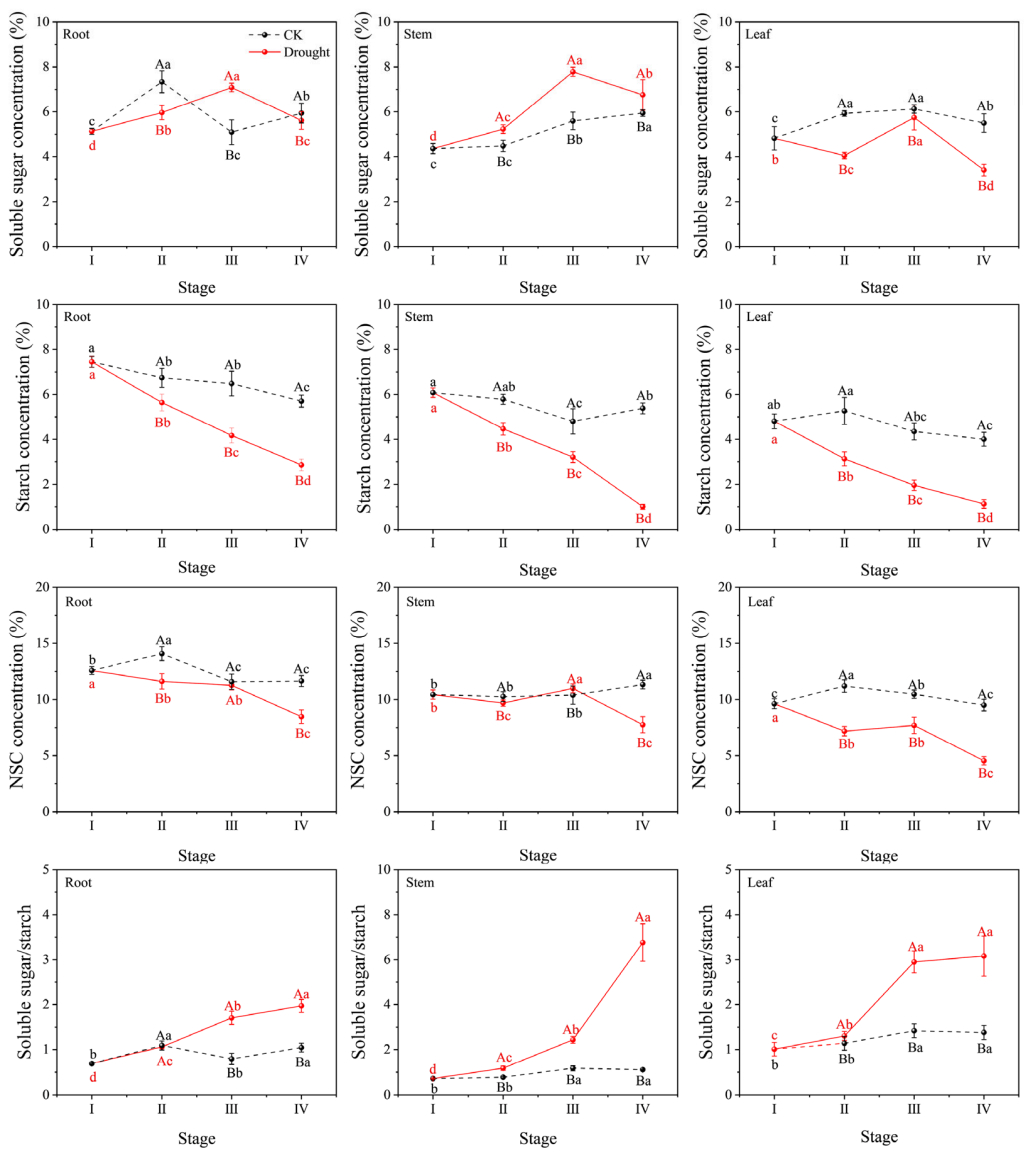
NSC concentration and the ratio of soluble sugar to starch in different parts of *Phoebe bournei* at various drought stages. Different capital letters indicate significant differences between treatments at the same stage, while different lowercase letters indicate significant differences between stages within the same treatment (*p* < 0.05).

**Figure 6:**
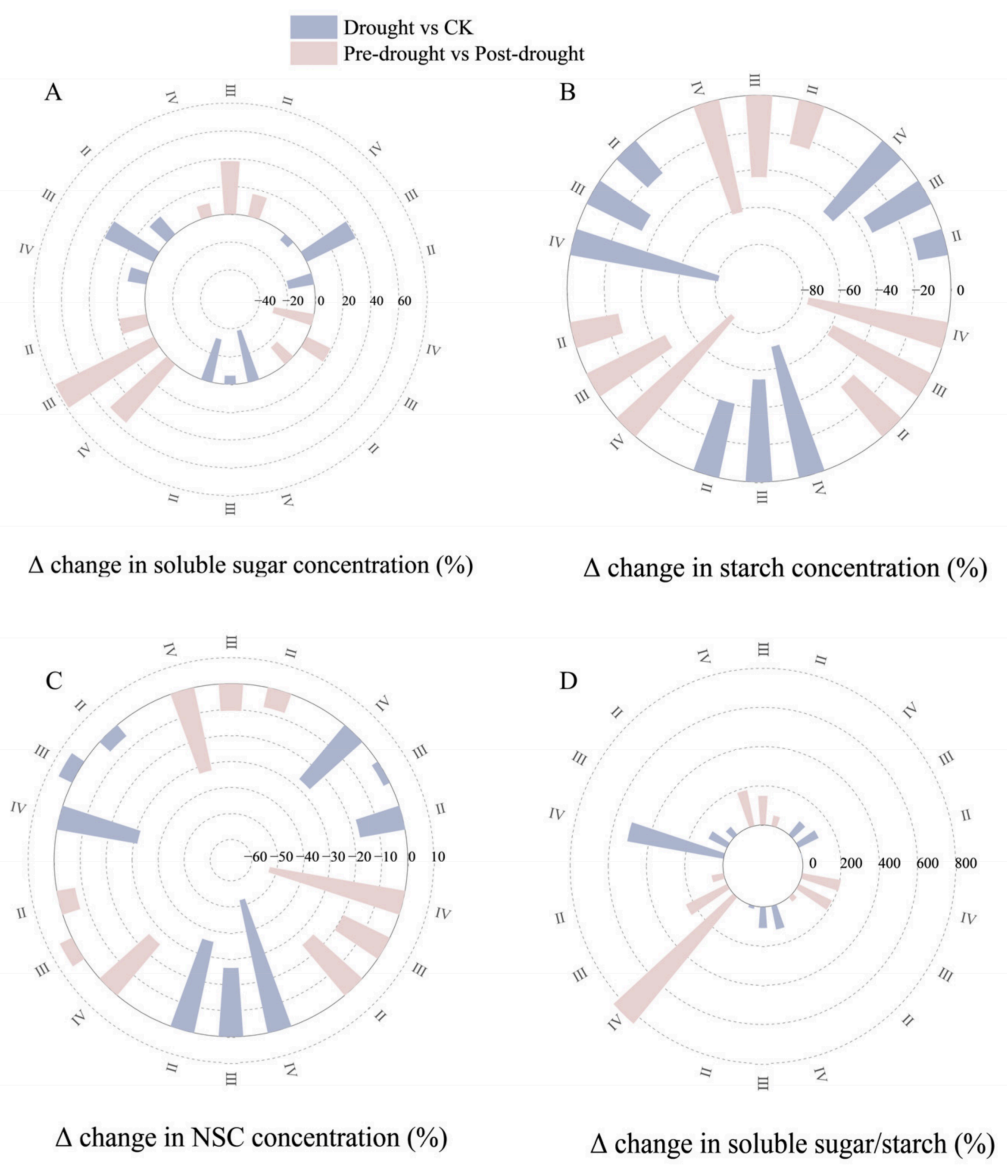
NSC concentration changes in different parts of *Phoebe bournei* at various drought stages. (**A**) Changes in the soluble sugar concentration of the drought-stressed group relative to the initial value and the concurrent control group at different stages; (**B**) changes in the starch concentration of the drought-stressed group relative to the initial value and the concurrent control group at different stages; (**C**) changes in the NSC concentration of the drought-stressed group relative to the initial value and the concurrent control group at different stages; (**D**) changes in the soluble sugar/starch of the drought-stressed group relative to the initial value and the concurrent control group at different stages. Positive values indicate an increase, while negative values indicate a decrease.

Root systems exhibited phasic fluctuations in soluble sugar concentrations in both experimental groups (Figure 5 and Figure 6). Control plants demonstrated significant carbohydrate accumulation during the zero photosynthetic rate phase, followed by sequential depletion during 100% leaf wilting and stem browning stages. Between-group comparisons revealed that drought-treated roots had significantly lower soluble sugar levels than controls at the zero photosynthetic phase, elevated concentrations during complete leaf wilting, and levels indistinguishable from controls at stem browning.

Stem tissues displayed pronounced inter-group differences in response. Control stems maintained a gradual ascending trajectory with non-significant variation at the zero photosynthetic phase and progressive increases during leaf wilting and stem browning stages. Conversely, drought-treated stems exhibited a distinct pattern characterized by initial accumulation followed by depletion, with all developmental phases showing significantly elevated soluble sugar concentrations relative to controls.

Leaf tissue analysis revealed contrasting temporal patterns between treatments. Control leaves manifested an initial increase, followed by a decrease in soluble sugar concentrations, whereas drought-treated leaves showed a consistent decline throughout the experimental period. A transient acceleration in carbohydrate accumulation occurred during leaf wilting in stressed plants, resulting in non-significant differences between groups at this specific physiological stage.

#### 3.4.2. Starch Concentration

Under intensifying drought conditions, starch concentrations in roots, stems, and leaves of drought-treated plants declined significantly, whereas control plants maintained relatively stable levels across all developmental stages. Notably, during the zero photosynthetic phase (II), leaf starch concentrations decreased most precipitously, with a greater reduction than in root and stem tissues, consistent with preferential mobilization of carbon reserves in photosynthetic organs to mitigate water deficit. As drought advanced to the leaf wilting phase (III) and the stem browning phase (IV), starch concentrations declined further across all organs, particularly in root systems, where depletion intensified. This pattern reflects a systemic imbalance in carbon allocation and the exhaustion of carbon reserves under drought conditions. The sequential starch depletion, initiated by pronounced leaf loss during early stress phases and progressing to root exhaustion, supports the pivotal role of carbon reserve exhaustion in drought-induced mortality of *P. bournei*. Early carbon loss in leaves likely accelerates overall physiological decline, highlighting the interconnectedness of organ-specific carbon dynamics in tree mortality pathways.

#### 3.4.3. Non-Structural Carbohydrate Concentration

Drought-treated plants exhibited consistent declines in non-structural carbohydrate concentrations across roots, stems, and leaves as stress intensified from Stage I to Stage IV, though with distinct patterns compared to controls. During the zero photosynthetic phase (II), drought-treated roots showed pronounced NSC depletion (8.82 ± 2.02%), which was significantly lower than that of contemporaneous controls. As stress progressed to irreversible stages of leaf wilting (III) and stem browning (IV), treated roots demonstrated accelerated NSC reduction. Stem NSC dynamics remained relatively stable in both groups, with controls exhibiting a decline-rise pattern, while treated plants showed a triphasic decline-rise-decline trajectory, ultimately falling significantly below control levels during the terminal stress phase. Leaf NSC variations proved most dramatic: control leaves displayed fluctuating concentrations, whereas treated leaves demonstrated sustained consumption from the initial stress stage, following a statistically significant linear decrease.

At the zero-photosynthetic phase, all organs in treated plants had lower NSC concentrations than their corresponding controls. By the stem browning phase, NSC reserves were severely depleted across all treated tissues: root NSC diminished to 67.36% of initial values and 72.76% of concurrent controls and stem NSC reduced to 74.33% of initial and 68.50% of control levels, while leaf NSC declined to 47.12% of initial and 47.73% of control concentrations. These results provide clear evidence that prolonged drought stress ultimately induces carbon starvation in *P. bournei* seedlings, characterized by systemic NSC depletion across all vegetative organs, with a particularly severe impact on photosynthetic tissues.

#### 3.4.4. Soluble Sugar/Starch

Drought treatment significantly altered the soluble sugar-to-starch ratio in all plant organs, with treated plants maintaining consistently higher ratios than controls throughout the experimental period. Root tissues exhibited the least fluctuation in these ratios, while stems and leaves showed more pronounced variations. Control plants demonstrated a pattern in root sugar-starch ratios (rise-decline-rise), with values slightly higher than those of treated plants during the zero-photosynthetic phase. However, treated plants maintained significantly higher ratios during both leaf wilting and stem browning phases. Stem tissues revealed significant between-group differences during critical stress phases. Control stems showed substantially lower ratios than treated stems at both the zero photosynthetic and complete leaf wilting stages, with particularly pronounced differences during the leaf wilting phase. Leaf tissues exhibited the most consistent drought-induced responses, with treated plants maintaining significantly higher soluble sugar-to-starch ratios than controls across all three stress phases: zero photosynthesis, leaf wilting, and stem browning.

These organ-specific responses are consistent with differential carbon allocation strategies under drought stress, with treated plants preferentially maintaining higher soluble sugar concentrations relative to starch reserves across all tissues. The sustained elevation of sugar-starch ratios in drought-treated plants, particularly during advanced stress phases, reflects increased osmotic adjustment demands and possible impairment of starch synthesis pathways under water deficit conditions.

## 4. Discussion

### 4.1. The Interaction Between Hydraulic Failure and Carbon Starvation: A Sequential Physiological Cascade

A central objective of this study was to elucidate the nature, sequence, and mechanistic details of the interaction between hydraulic failure and carbon starvation in driving drought-induced mortality in *P. bournei*. Our findings provide evidence that these two mechanisms are not independent parallel processes, but rather components of a tightly coupled, dynamically interacting physiological cascade. Hydraulic failure appears to be the initiating mechanism and proximate cause of death, while carbon starvation likely functions as a secondary amplifying factor that progressively undermines the capacity of the tree to resist hydraulic stress, thereby accelerating systemic failure. This interpretation aligns with the emerging consensus that drought-induced tree mortality is a complex syndrome involving multiple interacting physiological failures [13,31].

#### 4.1.1. Coordination During the Transition from Stage I to Stage II: Hydraulic Sensing Initiates, Carbon Starvation Germinates

In this initial phase, the tree senses declining soil water availability through root-to-shoot hydraulic and chemical signals and actively closes stomata to preserve hydraulic safety. This represents a strategic, regulated trade-off in which carbon gain is sacrificed to maintain the integrity of water transport [31,32]. Stomatal closure in *P. bournei* is primarily a hydraulic response driven by guard cell turgor loss as leaf water potential declines, rather than a carbon-mediated signal. The data show that stomatal conductance declined from 0.17 to 0.04 µmol m^−2^ s^−1^ by stage II while NSC concentrations were not yet significantly depleted (Table A1 in Appendix A). During this phase, growth is inhibited earlier and more sensitively than photosynthesis, leading to transient NSC accumulation as carbon sinks are reduced more rapidly than carbon sources [33]. Carbon starvation begins to germinate during this phase but is not yet the primary physiological constraint; rather, the plant is actively managing a strategic trade-off under hydraulic control.

#### 4.1.2. Amplification During the Transition from Stage II to Stage III: Hydraulic Failure Dominates as Carbon Starvation Gains Momentum

As drought severity exceeds the species’ physiological tolerance threshold, hydraulic dysfunction transitions from a regulated response to an unregulated, accelerating process. At Stage II, PLC had already reached 60%, showing that the majority of the xylem water transport capacity has been compromised. By Stage III, PLC approaches 77%, reflecting extensive embolism propagation that far outpaces any potential embolism repair. The near-complete cessation of photosynthetic carbon gain, combined with continued metabolic demand for respiratory maintenance metabolism, osmotic adjustment (evidenced by sustained elevation of the soluble sugar-to-starch ratio across all organs), and residual growth processes, initiates progressive NSC depletion. Critically, it is during this phase that the interaction between the two mechanisms likely becomes mutually reinforcing: declining carbon reserves impair energy-dependent embolism repair, as xylem refilling via phloem-driven water movement is an ATP-demanding process [34,35]; reduced carbohydrate availability limits osmolyte synthesis (including soluble sugars, proline, and other compatible solutes), compromising cellular osmotic adjustment and accelerating tissue dehydration; and energy limitation impairs antioxidant defense systems, permitting uncontrolled accumulation of reactive oxygen species that cause oxidative damage to membranes, proteins, and nucleic acids, further destabilizing cellular function. This phase represents the critical transition from reversible to irreversible damage, where carbon deficit begins to actively exacerbate hydraulic vulnerability. Our systematic re-watering experiments confirm this. At the zero-photosynthesis stage, with percent loss of conductivity of approximately 60% and predawn leaf water potential of approximately −3.4 MPa, 83% survival was observed upon re-watering, whereas the transition to irreversible damage occurs as percent loss of conductivity increases toward 77% and predawn leaf water potential declines below −5 MPa.

#### 4.1.3. Terminal Feedback During the Transition from Stage III to Stage IV: The Vicious Cycle Culminates in Mortality

The synergistic interaction appears to develop into a self-reinforcing cascade that drives the final decline to mortality. Severe xylem embolism exceeding 77% loss of conductivity secondarily impairs phloem translocation, as phloem transport depends on positive turgor pressure maintained by a continuous supply of water from the xylem [36,37]. When xylem water potential drops below a critical threshold, phloem turgor collapses, physically preventing redistribution of remaining reserves from storage organs to sites of high metabolic demand. This carbon compartmentation, where substantial reserves exist in roots at over 67% of initial values but are physically inaccessible, provides a plausible explanation for why NSC persisted in tissues at the point of death because catastrophic hydraulic failure terminated whole-plant function before those reserves could be mobilized. The resulting local energy deficit at embolism repair sites would further limit any residual capacity for embolism reversal and compromise active transport for osmotic adjustment. Meanwhile, cellular dehydration directly damages organelle membranes, disrupting electron transport and leading to uncontrolled reactive oxygen species production. This positive feedback loop operates as a self-reinforcing cascade; hydraulic failure suppresses photosynthesis and phloem transport, which drives local carbon depletion and systemic carbon compartmentation. These deficits impair repair capacity, osmotic adjustment, and oxidative stress control, thereby accelerating hydraulic deterioration. This cycle accounts for the rapid terminal decline in PLC from approximately 77% to approximately 98%. By the stem browning stage, with relative soil water content of approximately 18% and PLC exceeding 98%, mortality is certain irrespective of restored water availability.

### 4.2. Dynamic Changes in Percentage Loss of Conductivity and Hydraulic Vulnerability Threshold

Experimental data revealed an exponential increase in the PLC in *P. bournei* with declining soil moisture and plant water potential. During initial drought stages, plants maintained relatively stable water potential and low PLC through stomatal regulation. However, a distinct inflection point in the PLC curve occurred at approximately −3.4 MPa, beyond which PLC increased precipitously. This inflection point signifies a critical physiological transition, marking the onset of rapid hydraulic system deterioration. While hydraulic vulnerability curves vary across species, a common feature is an embolism threshold, beyond which gas emboli propagate rapidly within xylem conduits [38,39]. The inflection point at −3.4 MPa observed here is comparatively high (i.e., less negative) relative to many temperate or arid-zone trees. This confirms that *P. bournei* possesses a hydraulically vulnerable xylem, susceptible to extensive embolism under relatively moderate water stress. Anatomically, this vulnerability may be linked to xylem traits favoring efficiency over safety. Species with larger conduit diameters and pit membrane pores typically achieve higher hydraulic conductivity but exhibit reduced resistance to air-seeding and embolism spread [23]. As a species endemic to humid environments, the evolution of *P. bournei* appears to have prioritized water transport efficiency at the expense of hydraulic safety.

More importantly, re-watering experiments in this study identified an irreversible physiological threshold for mortality in *P. bournei*. When PLC exceeded 77% in the stem-browning group, even under restored soil water supply, the plants failed to recover transpirational and photosynthetic functions, leading to complete mortality within several days. This finding aligns closely with the lethal hydraulic failure thresholds (typically between PLC 50–90%) reported by Urli et al. for various angiosperm species [26,40]. Compared with the conifer *Pinus massoniana*, *P. bournei* appears more vulnerable. Multiple studies have shown that the P50 value (the water potential inducing 50% loss of conductivity) of P. massoniana generally ranges from −3.5 to −4.5 MPa [27,41]. This implies that at a water potential of −3.37 MPa, PLC in *P. massoniana* would likely remain relatively low, with its hydraulic system not yet entering a phase of rapid collapse. In contrast, at the same level of water stress, *P. bournei* already initiates severe embolism progression, and PLC rises sharply beyond this point. Such differences reflect the trade-offs in hydraulic safety strategies among species: angiosperms like *P. bournei* possess efficient yet vulnerable vessel systems, while gymnosperms like *P. massoniana* rely on safer but less efficient tracheid-based systems [42]. Furthermore, compared to another subtropical evergreen broad-leaved species, Cinnamomum camphora, whose greater drought tolerance implies stronger embolism resistance, *P. bournei* appears particularly susceptible [43]. Therefore, the relatively high inflection point water potential of *P. bournei* determines that its hydraulic system becomes the primary bottleneck limiting survival under extreme drought. These results also provide strong support for the theoretical framework proposed by McDowell et al., which posits that loss of hydraulic integrity is the direct cause of drought-induced mortality, taking precedence over carbohydrate depletion [15,44]. Once the water-transport network collapses, the plant loses the vital connection between root water sources and the photosynthetic machinery in leaves, making mortality inevitable.

### 4.3. Limitations and Future Research Directions

While this study provides insights into the physiological mechanisms of drought-induced mortality in *P. bournei*, several limitations should be noted. The experimental design used pot-based controlled conditions to simulate drought stress. However, these conditions may not fully reflect the complexities of natural environments. In natural settings, seedlings can develop deeper root systems accessing moisture from greater soil depths, potentially delaying the onset of lethal hydraulic thresholds. Additionally, natural soils exhibit spatial heterogeneity in moisture, nutrients, and microbial communities that may modulate drought responses in ways not captured by our homogeneous pot substrate. The restricted root growth and lack of natural soil processes in pots may limit direct comparisons to field conditions. The investigation also focused only on drought stress, without considering interactions with other environmental variables. In particular, co-occurring heat stress, which is increasingly common under climate change, may accelerate hydraulic failure and carbon depletion beyond the rates observed under drought alone. Biotic interactions, including pest and pathogen pressure that typically intensifies in drought-stressed trees, were not addressed in this study. Furthermore, our 51-day experiment captured only acute drought responses, whereas long-term acclimation processes such as changes in xylem anatomy, root-to-shoot allocation, and osmotic adjustment capacity may alter mortality thresholds in plants subjected to chronic stress.

Future research should prioritize integrated field studies that examine drought responses under natural environmental conditions. Such investigations would provide a more comprehensive understanding of mechanisms of tree mortality in realistic ecological contexts. Additionally, multifactorial experiments examining interactions between drought and other environmental stressors are essential for developing predictive models of tree mortality under climate change scenarios.

## 5. Conclusions

This study provides evidence that drought-induced mortality in *P. bournei* seedlings follows a sequential, staged physiological decline driven by the interacting deterioration of hydraulic and carbon metabolic functions. Our results indicate that hydraulic failure is the primary and proximate cause of mortality in this species: severe xylem embolism (PLC exceeding 77%) renders the water transport system non-functional, physically decoupling roots from leaves and making death inevitable regardless of residual carbon reserve status. Progressive NSC depletion, while not the direct trigger of mortality, likely contributes to the gradual loss of physiological resilience by impairing osmotic adjustment, energy-dependent embolism repair, and antioxidant defense, thereby lowering the hydraulic threshold at which catastrophic failure occurs and reducing the capacity for post-drought recovery. The two mechanisms are thus closely linked in a sequential manner, with hydraulic failure acting as the terminal driver of death and carbon depletion serving as a predisposing factor that amplifies vulnerability over time. Future studies should incorporate direct measurements of phloem function, embolism repair dynamics, tissue viability, reactive oxygen species accumulation, vulnerability curves (P_50_), and field-based validation to further test the interaction model proposed here.

## Figures and Tables

**Table 1:** Summary of experimental design. Ψ_leaf_, leaf water potential; *K*_h_, hydraulic conductivity; NSC, non-structural carbohydrate; PLC, percentage loss of hydraulic conductivity; CK, control group.

Stage	Description	Days	Groups (n = 6)	Key Measurements
I	Baseline	Day 0	Baseline	Gas exchange, Ψ_leaf_, NSC, *K*_h_, PLC
II	Zero photosynthesis	15	CK + Drought + Re-watering	Gas exchange, Ψ_leaf_, NSC, *K*_h_, PLC + 30-day recovery monitoring
III	100% leaf wilting	36	CK + Drought + Re-watering	NSC, *K*_h_, PLC + 30-day recovery monitoring
IV	Stem browning	45–51	CK + Drought + Re-watering	NSC, *K*_h_, PLC + 30-day recovery monitoring

## Data Availability

The original contributions presented in the study are included in the article. Further inquiries can be directed to the corresponding authors.

## Appendix A

**Table A1:** Key physiological thresholds across drought stages in *P. bournei* seedlings.

Parameter	Stage I	Stage II	Stage III	Stage IV
**RSWC (%)**	89.05	59.66	27.48	18.23
**Ψpd (MPa)**	−0.40	−3.37	<−5.0 *	<−5.0 *
**Ψmd (MPa)**	−1.19	−4.19	<−5.0 *	<−5.0 *
**Pn (μmol m^−2^ s^−1^)**	8.99	0.12	/	/
**Cond (mol m^−2^ s^−1^)**	0.17	0.04	/	/
**PLC (%)**	11.10	60.18	77.45	98.06
**NSC status**	Baseline	Initial depletion; transient accumulation	Progressive depletion	Severely depleted
**Survival after re-watering**	/	83% (5/6)	33% (2/6)	0% (0/6)
**Recovery Pattern**	/	New buds (15 d)	A few axillary buds	Irreversible mortality

* Values exceeded the instrument measurement range (<−5 MPa).
